# Comprehensive study of instable regions in *Pseudomonas aeruginosa* and *Mycobacterium tuberculosis*

**DOI:** 10.1186/s12938-018-0563-8

**Published:** 2018-11-20

**Authors:** Dan Wang, Jingyu Li, Lusheng Wang

**Affiliations:** 1 Department of Computer Science, City University of Hong Kong, 83 Tat Chee Ave., Hong Kong, People’s Republic of China; 2 University of Hong Kong Shenzhen Research Institute, Shenzhen Hi-Tech Industrial Park, Nanshan District, Shenzhen, People’s Republic of China

**Keywords:** Pan-genome, Dispensable genome, Insertion, Deletion, Homologous recombination, Directed repeats, Transposase, Integrase

## Abstract

**Background:**

*Pseudomonas aeruginosa* is a common bacterium which is recognized for its association with hospital-acquired infections and its advanced antibiotic resistance mechanisms. *Tuberculosis*, one of the major causes of mortality, is initiated by the deposition of *Mycobacterium tuberculosis*. Accessory sequences shared by a subset of strains of a species play an important role in a species’ evolution, antibiotic resistance and infectious potential.

**Results:**

Here, with a multiple sequence aligner, we segmented 25 *P. aeruginosa* genomes and 28 * M. tuberculosis* genomes into core blocks (include sequences shared by all the input genomes) and dispensable blocks (include sequences shared by a subset of the input genomes), respectively. For each input genome, we then constructed a scaffold consisting of its core and dispensable blocks sorted by blocks’ locations on the chromosomes. Consecutive dispensable blocks on these scaffold formed *instable regions*. After a comprehensive study of these instable regions, three characteristics of instable regions are summarized: instable regions were short, site specific and varied in different strains. Three DNA elements (directed repeats (DRs), transposons and integrons) were then studied to see whether these DNA elements are associated with the variation of instable regions. A pipeline was developed to search for DR pairs on the flank of every instable sequence. 27 DR pairs in *P. aeruginosa* strains and 6 pairs in *M. tuberculosis* strains were found to exist in the instable regions. On the average, 14% and 12% of instable regions in *P. aeruginosa* strains covered transposase genes and integrase genes, respectively. In *M. tuberculosis* strains, an average of 43% and 8% of instable regions contain transposase genes and integrase genes, respectively.

**Conclusions:**

Instable regions were short, site specific and varied in different strains for both *P. aeruginosa* and *M. tuberculosis*. Our experimental results showed that DRs, transposons and integrons may be associated with variation of instable regions.

**Electronic supplementary material:**

The online version of this article (10.1186/s12938-018-0563-8) contains supplementary material, which is available to authorized users.

## Background

Tettelin firstly coined the term pan-genome in his research of *Streptococcus agalactiae* [1] more than a decade ago. Pan-genome describes the union of genomes in a clade of interest, including core and dispensable genome. Core genome includes sequences shared by all genomes of interest while dispensable genome is the intersection of a subset of the genomes of interest. Core genome of a clade are typically responsible for the major phenotypic traits and basic aspects of the biology of this clade while dispensable genome contributes to the species diversity and persistence in a particular environment [2]. Identification and study of dispensable genome is essential for better understanding of a species’ evolution, niches adaptation, antibiotic resistance, infectious potential and colonization of a new host. Pan-genome of closely related strains can be obtained at the nucleotide sequence level using multiple whole-genome alignment tools.

Bacterial genomes are dynamic on the evolutionary time scale. The existence of dispensable genome of a bacterial species may arise from genome rearrangement events such as insertions or deletions (indels), or from the activities of mobile DNA elements such as transposons or integrons.

Several previous studies show that indels can be mediated by directed repeats (DRs) [3, 4]. And these DR-mediated indel events are named homologous recombination in which DNA strands are exchanged between a pair of similar or identical sequences. Sequences flanked by a pair of DRs can be deleted from the chromosomes or be inserted into new chromosomes, for example, numerous horizontally transferred genes are integrated into their new host chromosomes through homologous recombination [5]. The estimated minimal length of homologous sequences which are necessary for the recombination process is between 20 and 100 bp [6].

Existence of dispensable genome can also result from the activities of mobile DNA elements, such as transposons and integrons. There are two major ways for transposable genetic elements to move from one locus to another within or between genomes. One involves passage through an RNA intermediate prior to synthesis of a DNA copy while the other is limited uniquely to DNA intermediates. For both types of elements, recombination reactions involved in integration are carried out by element-specific enzymes. These are called transposase (Tnp) in the case of DNA elements and integrase in the case of the best-characterized RNA elements, the retroviruses and retrotransposons [7].

In our study, we analyzed 25 *Pseudomonas aeruginosa* and 28 *Mycobacterium tuberculosis* complete genomes downloaded from the NCBI GenBank database, respectively. Genomic sequences were segmented into core blocks (include sequences shared by all genomes) and dispensable blocks (include sequences shared by a subset of all genomes) by using a multiple sequence aligner named Mugsy [8]. Consecutive dispensable blocks flanked by a pair of core blocks formed an *instable region* (InsR) and this pair of core blocks are the *insertion site* for this instable region. DNA sequences within a instable region are called *instable sequences*. For the 28 *M. tuberculosis* strains (see Additional file 1: Table S5 for more details), we conducted the same experiment as *P. aeruginosa* strains and achieved 28 *M. tuberculosis* scaffolds.

## Results

In this section, we showed three characteristics of instable regions and illustrated two kinds of mechanisms for the insertion and deletion of instable regions based on *P. aeruginosa* and *M. tuberculosis* genomes.

### Characteristics of instable regions

We conducted a comprehensive study on instable regions in both *P. aeruginosa* and *M. tuberculosis* strains and found three characteristics of instable regions: instable regions were short, site specific and varied in different strains.

#### Instable regions are short

**Fig. 1:**
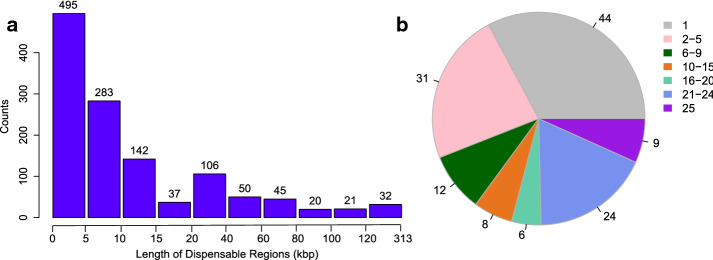
**a** Histogram for the length of instable regions (kbp) in *P. aeruginosa* strains. **b** Distribution of the 134 insertion sites in the 25 *P. aeruginosa* strains. 44 insertion sites exist in one strain, 31, 12, 8, 6, and 24 insertion sites exist in 2 to 5 strains, 6 to 9 strains, 10 to 15 strains, 16 to 20 strains and 21 to 24 strains, respectively, 9 insertion sites exist in 25 strains

Among the 25 *P. aeruginosa* strains, the number of instable regions was from 44 to 57 and the total lengths of instable regions were from 691,941 to 1,794,274 bps which accounted for 11% to 24% of their respective whole genome (see Additional file 1: Table S2). There were a total of 1231 instable regions among the 25 strains. The median and average length of these 1231 regions were 6.6 kbps and 20.9 kbps, respectively. The histogram for the length (in bp) of these 1231 instable regions is in Fig. 1a. We found that 40.2% of instable regions were shorter than 5 kbp, 77.7% of instable regions were shorter than 20 kbp and only 2.6% of instable regions are longer than 120 kbp (see Fig. 1a).

**Fig. 2:**
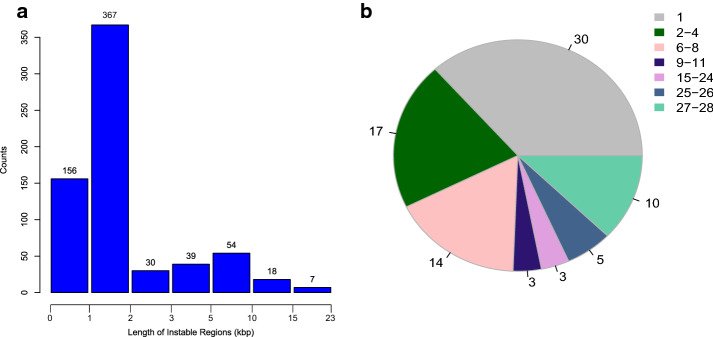
**a** Histogram for the length of instable regions (kbp) in * M. tuberculosis* strains. **b** Distribution of the 82 insertion sites in the 28 * M. tuberculosis* strains. 30 insertion sites exist in one strain, 17, 14, 3, 3, 5 and 10 insertion sites exist in 2 to 4 strains, 6 to 8 strains, 9 to 11 strains, 15 to 24 strains, 25 to 26 strains and 27 to 28 strains, respectively

In the 28 *M. tuberculosis* strains, the total amount of instable regions varied from 18 to 29. The total lengths of instable regions were from 40,009 to 71,578 bp which accounted for 0.91% to 1.62% of their respective whole genome (see Additional file 1: Table S5). A total of 671 instable regions existed in the 28 *M. tuberculosis* strains. The median and average length of these 671 regions were 1300 bp and 2343 bp, respectively. We plotted the histogram for the length (in bp) of these 671 instable regions (see Fig. 2a). 77.9% of instable regions were shorter than 2 kbp, 96.3% of instable regions were shorter than 10 kbp and only 1.04% of instable regions are longer than 15 kbp (see Fig. 2a).

#### Instable regions are site-specific

For 25 *P. aeruginosa* strains, 451 core blocks could be generated in the scaffolds. If the orders of these core blocks are consistent in the 25 scaffolds, there will be 450 distinct pairs of adjacent core blocks which can be the potential insertion sites for instable regions. But in fact, the orders of core blocks were different in the 25 scaffolds, which may result from genome rearrangements. As a consequence, we found a total of 506 distinct pairs of adjacent core blocks which can be the potential insertion sites for instable regions. Among the 506 pairs of adjacent core blocks, 134 pairs of core blocks are found between which instable regions existed (the block IDs of these 134 pairs of core blocks are listed in Additional file 1: Table S3). For these 134 pairs of core blocks, 90 of them were insertion sites in two or more strains while 44 of them were insertion sites in only one strain (see Fig. 1b). There were 9 pairs of core blocks which were insertion sites in all the 25 strains (see Fig. 1b).

As for *M. tuberculosis* strains, there were 107 distinct pairs of adjacent core blocks which can be the potential insertion sites of instable regions. However, 82 pairs of core blocks between which instable regions really existed. Within the 82 pairs of core blocks, 52 insertion sites were in two or more strains while 30 existed in only one strain in all the 28 *M. tuberculosis* strains (see Additional file 1: Table S6).

#### Instable regions vary in different strains

**Fig. 3:**
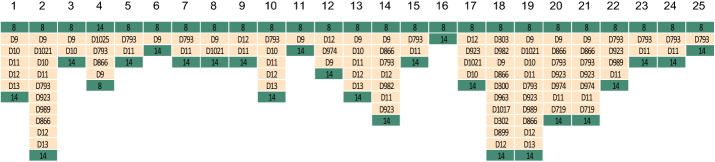
Instable regions between Core Block 8 and 14 in the 25 *P. aeruginosa* strains. The ID of each strain is labeled above its respective column. Column x is the partial scaffold between Core Block 8 and 14 in Strain x, where x = 1, 2,..., 25. Core blocks and dispensable blocks are respectively represented by green and yellow rectangular boxes with block IDs written on them

Between the same pair of core blocks, the inserted instable region varied a lot in different strains. For example, between Block 8 and 14, the instable regions in the 25 *P. aeruginosa* strains were different (see Fig. 3). For *P. aeruginosa* strains, among the 90 insertion sites which existed in two or more strains, instable regions in 57 insertion sites were varied in different strains (the block IDs of these 57 insertion sites are in Additional file 1: Table S3). In *M. tuberculosis* strains, instable regions in 25 (out of 52) insertions sites are varied in different strains (see Additional file 1: Table S6).

### Mechanisms for the variation of instable regions

Due to the large variation of instable regions, we focused on directed repeats (DRs), transposase genes and integrase genes. Those DNA elements may contribute to the variation of instable regions.

#### Indels mediated by directed repeats (homologous recombination)

We developed a pipeline to find directed repeat pairs (for the explanation of DRs, see “Methods” section) which were on the flank of an instable sequence and applied it on the 25 *P. aeruginosa* strains and 28 *M. tuberculosis* strains, respectively. After achieving DRs by using our pipeline, we added those DRs into the 25 *P. aeruginosa* scaffolds and 28 *M. tuberculosis* scaffolds respectively according to DRs’ positions in the chromosomes (see the scaffolds in Additional file 1: Tables S1 and S4, DRs are represented by R plus an ID number).

After studying the scaffolds with DRs, we found 27 pairs of DRs in *P. aeruginosa* strains and 6 pairs of DRs in *M. tuberculosis* strains existing in the instable regions.

**Fig. 4:**
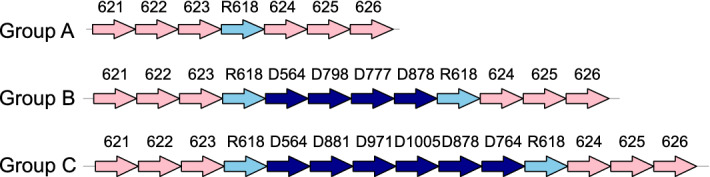
Insertion of instable regions mediated by R618 in *P. aeruginosa* strains. The three rows are the partial scaffolds (from Block 621 to 626) of strains in Group A, B and C. Blocks are represented by arrows. Core blocks are in pink, dispensable blocks are in dark blue and Repeat R618 is in light blue. The number above an arrow is the ID of the corresponding block

Figure 4 one example pair of DRs found in *P. aeruginosa* which may mediate the change of the instable region between Core Block 623 and 624 (see Fig. 4 and the DR ID is R618). In Fig. 4, the 25 strains are divided into three groups (Group A, B, and C) according to their scaffolds between Core Block 623 and 624. Group A includes Strain 1, 3, 5, 7, 9, 10, 11, 12, 13, 15, 16, 17, 20, 21 and 25, Group B includes Strain 2, 4, 14, and 23 and Group C includes Strain 8 only. In the strains of Group A, there was only one copy of R618 between Block 623 and 624 while in the strains of Group B and C, there was an instable region flanked by a pair of R618 between Block 623 and 624 (see Fig. 4). The instable regions in Group B and C were different: the instable region of Group B contained dispensable blocks D564, D798, D777 and D878 while the instable region of Group C contained D564, D881, D971, D1005, D878 and D764 (see Fig. 4). We believed the pair of R618 mediated the insertion of the instable regions in Group B and C because the existence of the two copies of R618 made sure the ends of Block 623 and 624 remained unchanged before and after the insertion of the instable region. For every achieved DR pair *Rn*, we showed the 25 strains’ partial scaffolds covering *Rn* in Additional file 1: Table Rn (where n is the ID of this DR pair).

**Fig. 5:**
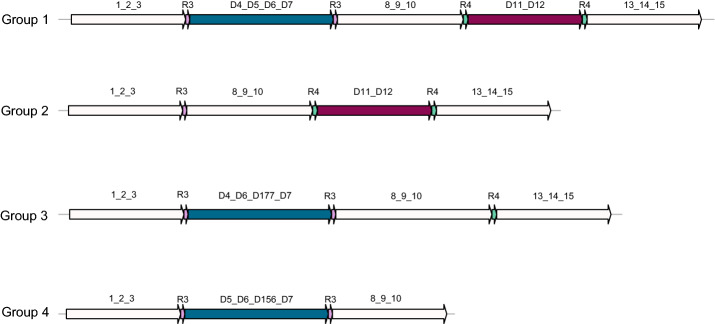
Two example DRs found in * M. tuberculosis* strains. R3 and R4 are two DRs found in *M. tuberculosis* strains. 28 *M. tuberculosis* strains are divided into four groups, Group 1–4. Group 2 includes Strain 6 and 9, Group 3 includes Strain 21 and 28, Group 4 includes Strain 14 and Group 1 includes the remaining strains. R3 is related to the insertion or deletion of the blue region and R4 is related to that of the red region

Two example pairs of DRs (see Fig. 5 and DR IDs are R3 and R4) found in *M. tuberculosis* strains is demonstrated in Fig. 5. According to the existence of the blue and red regions, the 28 *M. tuberculosis* strains are divided into four groups (Group 1–4, see Fig. 5). Group 2 includes Strain 6 and 9, Group 3 includes Strain 21 and 28, Group 4 includes Strain 14 and Group 1 includes the remaining strains. The insertion or deletion of the blue region is mediated by R3 and that of the red region is mediated by R4.

The remaining DRs are shown in Additional file 1: Tables S1, S4 and Rn, where n is the ID of DR.

#### Transposase and integrase genes

We added the genes whose products are integrase and transposase into the scaffolds for all the strains except *P. aeruginosa* Strain 23, 15 and 24 which lacks annotation information (see the scaffolds in Additional file 1: Tables S1 and S4, TNP and IN represent transposase and integrase genes, respectively). We then further studied the correlations between these genes and instable regions. In *P. aeruginosa* strains, we found that more than 60% of integrase and transposase genes were located inside the instable regions in all the strains except Strain 25 (see Table 1, Column P1 and P2). In *P. aeruginosa* Strain 3,4,5,6,7,8,9,10,11,12,14,17,20 and 21, all genes which encode to be transposase were in instable regions and for Strain 20, 21 and 16, all integrase genes were inside the instable regions (see Table 1, Column P1 and P2). We found that, in *P. aeruginosa* strains, 2%–30% covered integrase genes (see Column P4 of Table 1) and 2% to 42% of instable regions covered transposase genes (see Column P3 of Table 1). On average, 92% of transposase genes were within instable regions, while 85% of integrase genes were within instable regions, 14% of instable regions cover transposase genes and 12% of instable regions cover integrase genes in *P. aeruginosa* strains (see the last row in Table 1).

**Table 1:** Genes of transposase and integrase in instable regions of *P. aeruginosa* Strain 1 to 25

P1*	P2*	P3*	P4*
81% (34/42)	83% (10/12)	27% (15/56)	13% (7/56)
96% (48/50)	92% (11/12)	28% (14/50)	14% (7/50)
100% (6/6)	88% (7/8)	11% (5/47)	13% (6/47)
100% (17/17)	67% (6/9)	17% (9/52)	12% (6/52)
100% (5/5)	88% (7/8)	9% (4/45)	11% (5/45)
100% (1/1)	71% (5/7)	2% (1/48)	10% (5/48)
100% (12/12)	75% (3/4)	16% (7/44)	7% (3/44)
100% (2/2)	83% (5/6)	4% (2/52)	8% (4/52)
100% (13/13)	90% (9/10)	13% (6/45)	13% (6/45)
100% (25/25)	89% (8/9)	20% (11/54)	11% (6/54)
100% (8/8)	88% (7/8)	6% (3/51)	14% (7/51)
100% (15/15)	92% (12/13)	9% (4/47)	13% (6/47)
80% (32/40)	83% (10/12)	27% (15/56)	13% (7/56)
100% (5/5)	91% (10/11)	9% (4/47)	11% (5/47)
92% (11/12)	100% (10/10)	11% (5/47)	13% (6/47)
100% (8/8)	83% (5/6)	11% (5/47)	9% (4/47)
95% (21/22)	84% (21/25)	18% (8/45)	24% (11/45)
86% (68/79)	90% (26/29)	42% (24/57)	30% (17/57)
100% (1/1)	100% (1/1)	2% (1/45)	2% (1/45)
100% (1/1)	100% (1/1)	2% (1/45)	2% (1/45)
69% (40/58)	82% (9/11)	29% (14/49)	14% (7/49)
17% (1/6)	50% (1/2)	2% (1/45)	2% (1/45)
92%	85%	14%	12%

P1 is the percentage of Tnp genes within InsRs (no. of Tnp in InsRs/total no. of Tnp). P2 is the percentage of IN genes within InsRs (no. of IN in InsRs/total no. of IN). P3 is the percentage of InsRs which covers Tnp genes (no. of InsRs which covers Tnp Genes/total no. of InsRs). P4 is the percentage of InsRs which covers IN genes (no. of InsRs which covers IN Genes/total no. of InsRs)

Table 2 shows the situations of integrase and transposase genes in instable regions of *M. tuberculosis* Strain 1 to 28. We found that in *M. tuberculosis* strains, on average, 45% of transposase genes were within instable regions and 28% of integrase genes were within instable regions. On average, 43% and 8% of instable regions in *M. tuberculosis* strains covered transposase and integrase genes respectively.

**Table 2:** Genes of transposase and integrase in instable regions of *M. tuberculosis* Strain 1 to 28

P1*	P2*	P3*	P4*
42% (14/33)	29% (2/7)	35% (9/26)	8% (2/26)
29% (10/35)	17% (1/6)	20% (5/25)	4% (1/25)
40% (14/35)	33% (2/6)	21% (6/29)	7% (2/29)
64% (32/50)	14% (1/7)	71% (17/24)	4% (1/24)
34% (12/35)	0% (0/6)	21% (6/28)	0% (0/28)
57% (29/51)	17% (1/6)	77% (20/26)	4% (1/26)
35% (13/37)	38% (3/8)	32% (8/25)	12% (3/25)
70% (30/43)	43% (3/7)	77% (17/22)	9% (2/22)
33% (12/36)	14% (1/7)	24% (5/21)	5% (1/21)
32% (11/34)	25% (2/8)	33% (6/18)	11% (2/18)
35% (13/37)	38% (3/8)	31% (8/26)	12% (3/26)
42% (15/36)	67% (2/3)	35% (9/26)	8% (2/26)
33% (12/36)	38% (3/8)	30% (6/20)	15% (3/20)
31% (11/36)	14% (1/7)	20% (4/20)	5% (1/20)
65% (32/49)	14% (1/7)	67% (18/27)	4% (1/27)
45% (20/44)	38% (3/8)	59% (13/22)	14% (3/22)
72% (33/46)	43% (3/7)	78% (18/23)	9% (2/23)
41% (14/34)	17% (1/6)	24% (6/25)	4% (1/25)
40% (14/35)	17% (1/6)	23% (6/26)	4% (1/26)
72% (34/47)	43% (3/7)	79% (19/24)	8% (2/24)
35% (13/37)	38% (3/8)	45% (9/20)	15% (3/20)
39% (14/36)	17% (1/6)	22% (6/27)	4% (1/27)
51% (20/39)	25% (2/8)	45% (9/20)	10% (2/20)
35% (11/31)	29% (2/7)	33% (6/18)	11% (2/18)
58% (32/55)	38% (3/8)	69% (18/26)	12% (3/26)
40% (14/35)	17% (1/6)	24% (6/25)	4% (1/25)
41% (14/34)	29% (2/7)	35% (9/26)	8% (2/26)
62% (31/50)	43% (3/7)	69% (18/26)	0% (3/26)
45%	28%	43%	8%

**Fig. 6:**
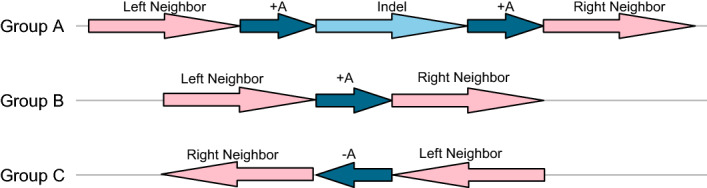
Visualization of the 25 scaffolds. Each row represents the scaffold of the corresponding strain. Horizontal colored blocks are core blocks and white blocks are dispensable blocks. Each color represents a distinct set of core blocks. Integrase, transposase genes and DRs genes are represented by red, green and blue vertical bars

**Fig. 7:**
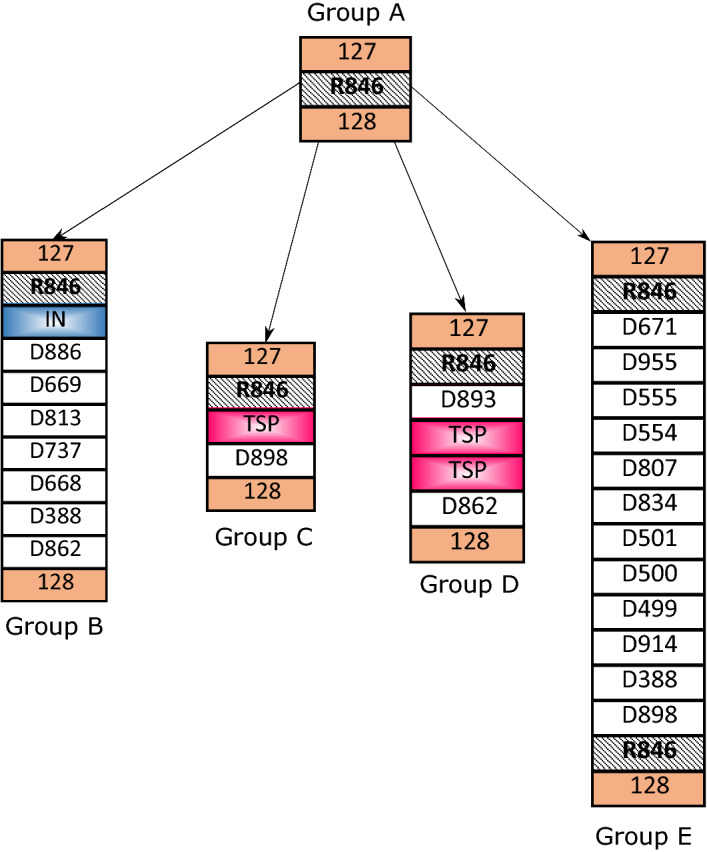
Visualization of the 28 *M. tuberculosis* scaffolds

To sum up, we drew 28 scaffolds for *M. tuberculosis* strains and 25 scaffolds for *P. aeruginosa* strains. These scaffolds are composed by core blocks, dispensable blocks, transposase, directed repeats, and integrase genes and sorted by their positions in the strains’ chromosomes (see Additional file 1: Tables S1 and S4). We plotted Figs. 6 and 7 to visualize the *P. aeruginosa* scaffolds and *M. tuberculosis* scaffolds respectively. From Figs. 6 and 7, we can see that transposase, directed repeats and integrase genes are in high correlation with instable regions.

## Methods

In this section, we introduced how gene scaffolds for *P. aeruginosa* and *M. tuberculosis* strain sets are established and the pipeline details for finding DRs that will mediate insertion and deletion of instable blocks. In the end, we explained the method for finding DNA elements such as transposases and integrases.

### Building scaffolds and finding instable regions

Sequences in input genomes were segmented into core blocks, dispensable blocks and strain-specific blocks by Mugsy [8], a multiple sequence aligner. In this work, we omitted small blocks which is shorter than 1000 bp and strain-specific blocks. To distinguish between different blocks, we labeled each core block with an distinct ID number and labeled each dispensable block with “D” plus an ID number. For each input genome, we then built a scaffold which consisted of the IDs of core and dispensable blocks sorted by starting positions on the chromosomes (see Additional file 1: Table S1). In the scaffolds, consecutive dispensable blocks formed instable regions and we then did a comprehensive study on these instable regions.

### Finding insertions and deletions mediated by DRs

**Fig. 8:**
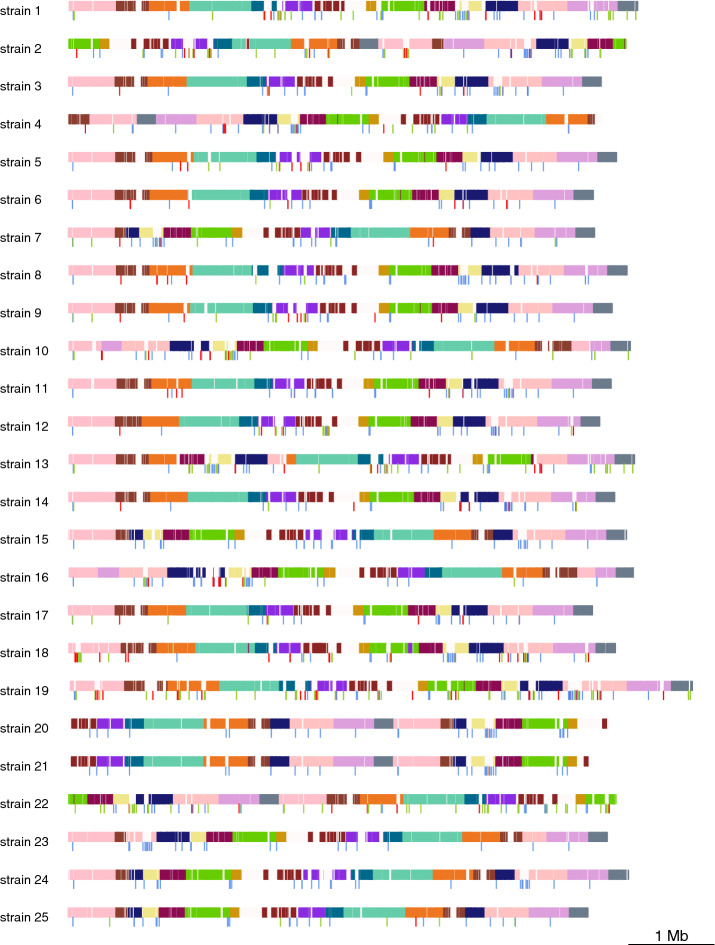
Layout of the strain groups before and after repeat-mediated insertion or deletion. In this figure, “+” and “−” represent the directions of segments. When deletion of the region in sky blue occurs on group B and group C, sky blue region is removed together with one piece of repeat in dark blue (+A) compared with group A, but the left neighbor and right neighbor in pink keep unchanged

In order to figure out directed repeats that are related to instable regions, we developed a pipeline based on BLAST [9] alignment method that can find the similar structure which is shown in Fig. 8. For each instable block, act the following four procedures to find out a reasonable pair of repeats in the same direction that may be associated with the insertion or deletion of one instable region.

Firstly, regarding each instable block (define as *InsB*) on each strain (define as strain *A1*), cut 10,000 base pairs from the left side of *InsB* and name it as *reA*. Define the start position of *reA* as *reA_s* and the end position as *reA_e*.

Secondly, use BLAST to do alignment between *reA* and *target segment*. *Target segment* means the gene segment that starts from *reA_e* and ends on the tail of strain *A1*. The corresponding BLAST alignment outcome shows as a chart which has a set of DNA segment pairs (name this set of gene pairs as *candA_list* and the members in it as *candA*s), and each of them is situated at both sides of *InsB*, illustrating that these gene segment pairs could be the possible reason of *InsB*’s insertion or deletion. Each line of *candA_list* contains four positions, left *candA*’s start position, left *candA*’s end position, right *candA*’s start position and right *candA*’s end position. We define them as *candAL_s*, *candAL_e*, *candAR_s* and *candAR_e*, respectively. For convenience, gene segment from *candAL_e* to *candAR_s* is named as *INDEL*, segment from *candAL_s − 10,000* to *candAL_s* is named as *neighborL* and segment from *candAR_e* to *candAR_e + 10,000* is named as *neighborR*.

Thirdly, in order to find out which component in *candA_list* are linked to *InsB* insertion or deletion with the highest possibilities, we searched each *candA* together with its corresponding *neighborL* and *neighborR* on those strains that lack *InsB*. If *neighborL–candA–neighborR* structure can be found on those strains and *neighborL–candA–InsB–candA–neighborR* structure can be found on strain *A1* simultaneously, set this *candA* as *RepeatA*.

Lastly, for further demonstration, we check the *INDEL* region on all 25 strains. If *INDEL* region always appears together with a pair of *RepeatA* on both sides, *RepeatA* can be regarded as a reasonable repeat that causes *INDEL* region’s insertion or deletion.

Our approach checked all the instable blocks in the .maf file and found out all the possible *RepeatA*s that may be the reason of gene indels. While applying BLAST package into our project, we set the E-value threshold as $10^{-10}$.

### Finding transposase and integrase genes

For the two datasets, we got the positions of genes which are encoded as integrase or transposase from the annotation files in GFF format. These annotations can be accessed on NCBI. We then add these genes to their strains’ scaffolds according to their positions (see Additional file 1: Tables S1 and S4).

**Fig. 9:**
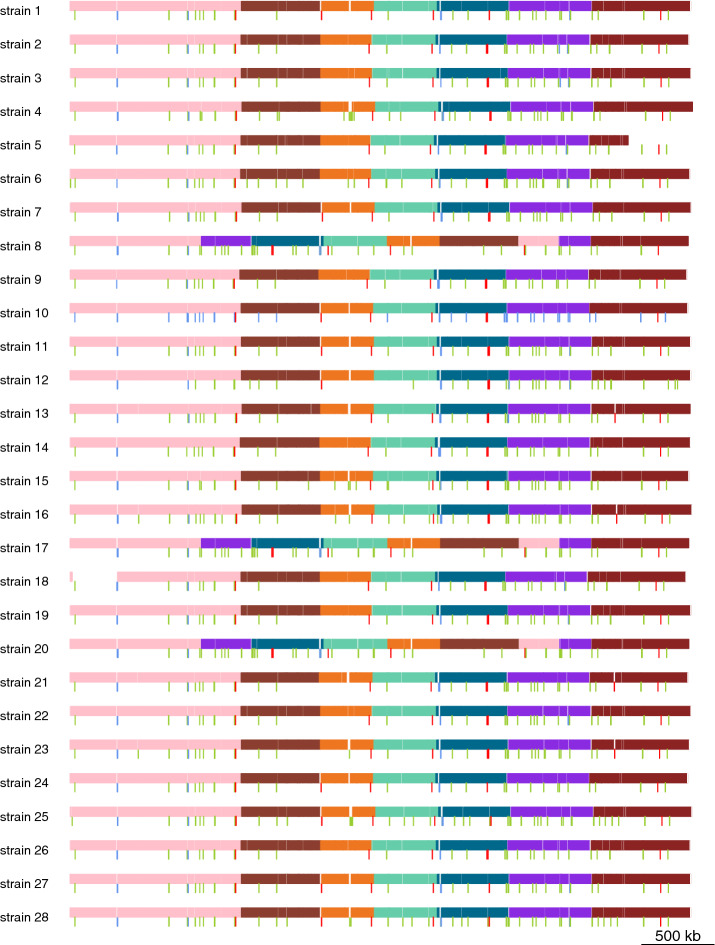
Evolution of strains through different mechanisms. The five columns are the partial scaffolds (from Block 127 to 128) of strains in Group A, B, C, D and E. Core blocks, dispensable blocks, DRs, transposase and integrase genes are represented by rectangular boxes in orange, white, gray, deep pink and blue, respectively. Group A includes Strain 1, 2, 3, 5, 6, 8, 9, 10, 11, 12, 13, 14, 15, 16, 19, 22, 24 and 25. Group B includes Strain 4. Group C includes Strain 7 and 18. Group D includes Strain 17, 23. Group E includes Strain 20 and 21

## Discussion

With the scaffolds consisting of core blocks, dispensable blocks, DRs, transposase genes and integrase genes, one can clearly observe the variations of an instable region among strains and the possible mechanisms for each variation. As illustrated in Fig. 9, the 25 *P. aeruginosa* strains can be stratified into five groups (A–E) based on their instable regions between Core Block 127 and 128. Strains of Group B, C, D and E may evolve from strains of Group A through different DNA elements: the insertion of Block D886 to Block D862 in strains of Group B may be mediated by the integrase gene (blue); the insertion of dispensable block D898 in Group C may be caused by the transposase gene (deep pink); the two transposase genes (deep pink) may lead to the insertion of dispensable block D893 and D862 in the strains of Group D; the insertion of Block D671 to Block D898 in strains of Group E may be mediated by the pair of R846.

Other genetic elements, recombinations or systems, such as ICEs [10], resolvases [11], invertases [12], Illegitimate recombination [13] and CRISPR–Cas systems [14, 15], may also contribute to the variation of instable regions. In our future work, we will consider these factors to explain more variations in genomes.

## Conclusions

Three characteristics of instable regions were concluded in both *P. aeruginosa* and *M. tuberculosis* strains: instable regions were short, site specific and varied in different strains. Three DNA elements (DRs, transposons and integrons) which may be associated with the variation of instable regions were studied. A pipeline was developed to automatically search for DR pairs on the flank of every instable sequence. We found 27 DR pairs in *P. aeruginosa* strains and 6 pairs in *M. tuberculosis* strains existing in the instable regions. Besides, on the average, 14% and 12% of instable regions in the 25 *P. aeruginosa* strains include transposase genes and integrase genes, respectively. In *M. tuberculosis* strains, an average of 43% and 8% of instable regions include transposase genes and integrase genes, respectively.

## Additional file

**Additional file 1: Table S1.** Sca_olds for the 25 *Pseudomonas aeruginosa* genomes. **Table S2.** The 25 *Pseudomonas aeruginosa* strains used for studying instable regions. **Table S3.** 134 distinct pairs of core blocks between which instable region existed in *Pseudomonas aeruginosa* strains. **Table S4.** Sca_olds for the 28 *Mycobacterium tuberculosis* genomes.** Table S5.** The 28 *Mycobacterium tuberculosis* strains used for studying instable regions. **Table S6.** 82 distinct pairs of core blocks between which instable region existed in *Mycobacterium tuberculosis* strains. **Table Rn.** For every achieved DR pair Rn, we showed the 25 *Pseudomonas aeruginosa* strains' partial sca_olds covering Rn, where n is the ID of this DR pair.

## Abbreviations

Tnp: transposase
IN: integrase
InsR: instable region
DR: directed repeat
